# The Causation of Peritonitis

**Published:** 1905-03-04

**Authors:** 


### THE CAUSATION OF PERITONITIS.

In their Erasmus Wilson lectures Mr. Leonard Dudgeon and Mr. Percy Sargent have made a valuable contribution to the knowledge of the intimate causation of peritonitis, that is, of its bacteriology.

It has long been known that a gross hæmorrhage into the peritoneal cavity, such, for instance, as that resulting from traumatic rupture of the liver or spleen, or from rupture of a tubal pregnancy, was followed by an inflammatory reaction of the peritoneum adjacent to the blood effused. This was thought to be due to some mysterious property inherent in infused blood, and was provisionally spoken of as "chemical peritonitis" in contradistinction from the admittedly bacteriological varieties. The authors have shown, as the result of an admirable series of observations, that effused blood in the peritoneum rapidly becomes infected with a white staphylococcus of very low virulence which is the real agent in the production of the observed inflammatory sequels.

In the matter of perforated gastric ulcers the authors succeeded in isolating a strepto-diplococcus from the edge of the ulcer in every one of their cases, and from the peritoneal exudate in a large proportion. This organism is very slightly pathogenic to rabbits, and the authors, while declining to claim for it a positive part in the causation of the disease, very fairly regard its constant presence as highly suggestive of its responsibility. Prognostically, the low virulence of the organism renders hopeful those cases in which it is found alone, while if the bacillus coli is present the outlook is made in the highest degree grave.

With regard to perforations of the gut in typhoid fever, it appears that the resulting peritonitis is quite seldom due to the bacillus typhosus but more commonly to the bacillus coli, or to staphylococci.

Some of their conclusions which bear on surgical practice are especially worthy of note. Thus, with regard to drainage of the peritoneum, they point out that in mild infections it is unnecessary, while in virulent infections drainage is impossible. The material used for drainage very soon becomes isolated by adhesions from the general peritoneal cavity, and merely provides a track which is open to secondary infection from the skin and external surroundings. And with regard to flushing out the peritoneum with large quantities of sterilised water or saline solutions, this is permissible in cases where there is no severe infection, as in peritoneal hæmorrhage from ruptured ectopic gestation, and in such cases may serve to wash out blood clots, and have other beneficial results, but in the presence of virulent infections the lavage is likely to cause a diffusion of the septic material and so be most detrimental. It is better in such cases to limit intra-abdominal manipulation so far as possible, and confine the cleansing process to dry swabbing.

